# Challenging diagnosis of drug reaction with eosinophilia and systemic symptoms (DRESS) in a patient with adult-onset Still disease

**DOI:** 10.1016/j.jdcr.2026.06.067

**Published:** 2026-07-10

**Authors:** Anusha M. Kumar, Jenny Lai, Lin J. He, George F. Murphy, Sotonye Imadojemu

**Affiliations:** a Department of Dermatology, Beth Israel Deaconess Medical Center, Boston, Massachusetts; b Harvard Medical School, Boston, Massachusetts; c Department of Dermatology, Brigham and Women’s Hospital, Boston, Massachusetts; d Department of Anatomical Pathology, University of Texas MD Anderson Cancer Center, Houston, Texas; e Department of Pathology, Brigham and Women’s Hospital, Boston, Massachusetts

**Keywords:** adult-onset Still disease, anakinra, complex medical dermatology, dermatopathology, drug hypersensitivity, drug reaction with eosinophilia and systemic symptoms

## Introduction

Adult-onset Still disease (AOSD) is a systemic autoinflammatory disorder with manifestations including spiking fevers, polyarthralgia, and cutaneous eruptions. The latter typically presents as evanescent erythematous macules or papules, often coincident with fevers, though persistent urticarial and lichenoid plaques or erythroderma have been reported.^1^ Treatment of AOSD involves NSAIDs and systemic corticosteroids for initial disease control, and biologic agents are increasingly used for maintenance.^2^ Systemic drug reactions with skin manifestations have been reported in patients with AOSD treated with IL-1 and IL-6 inhibitors, complicating the diagnosis of atypical eruptions in AOSD.^3^ Here, we present a case of a patient recently diagnosed with AOSD who developed a diffuse eruption concerning for a disease flare. The eruption was refractory to escalating immunosuppression including anakinra (IL-1 receptor antagonist). Histopathology solidified the diagnosis of drug hypersensitivity, with symptom resolution following anakinra withdrawal.

## Case report

**Fig 1:**
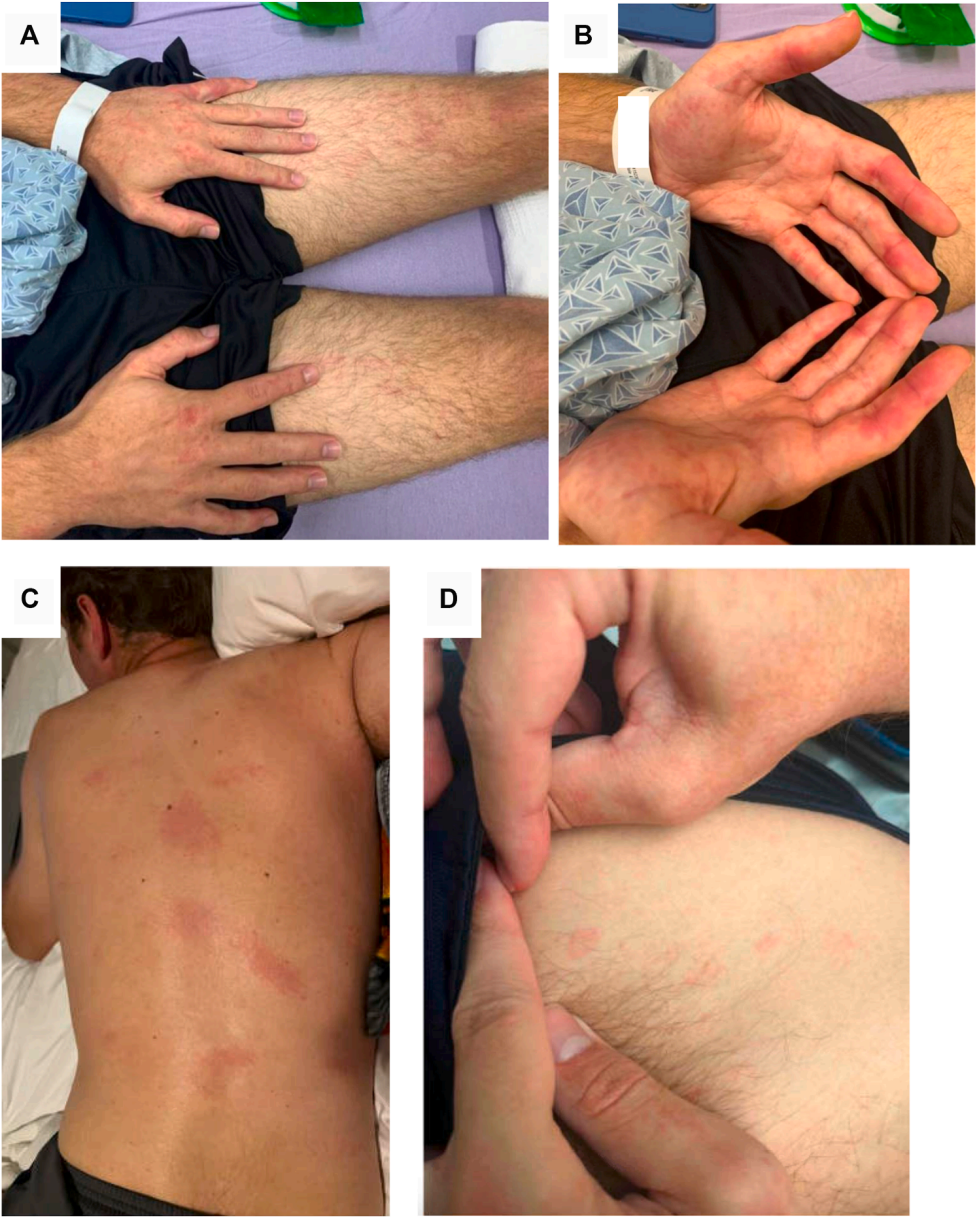
Evanescent urticarial papules and plaques at time of initial diagnosis of adult-onset Still’s disease (AOSD) **(A** and **B)** and re-presentation for macrophage activation syndrome **(C** and **D)**.

**Fig 2:**
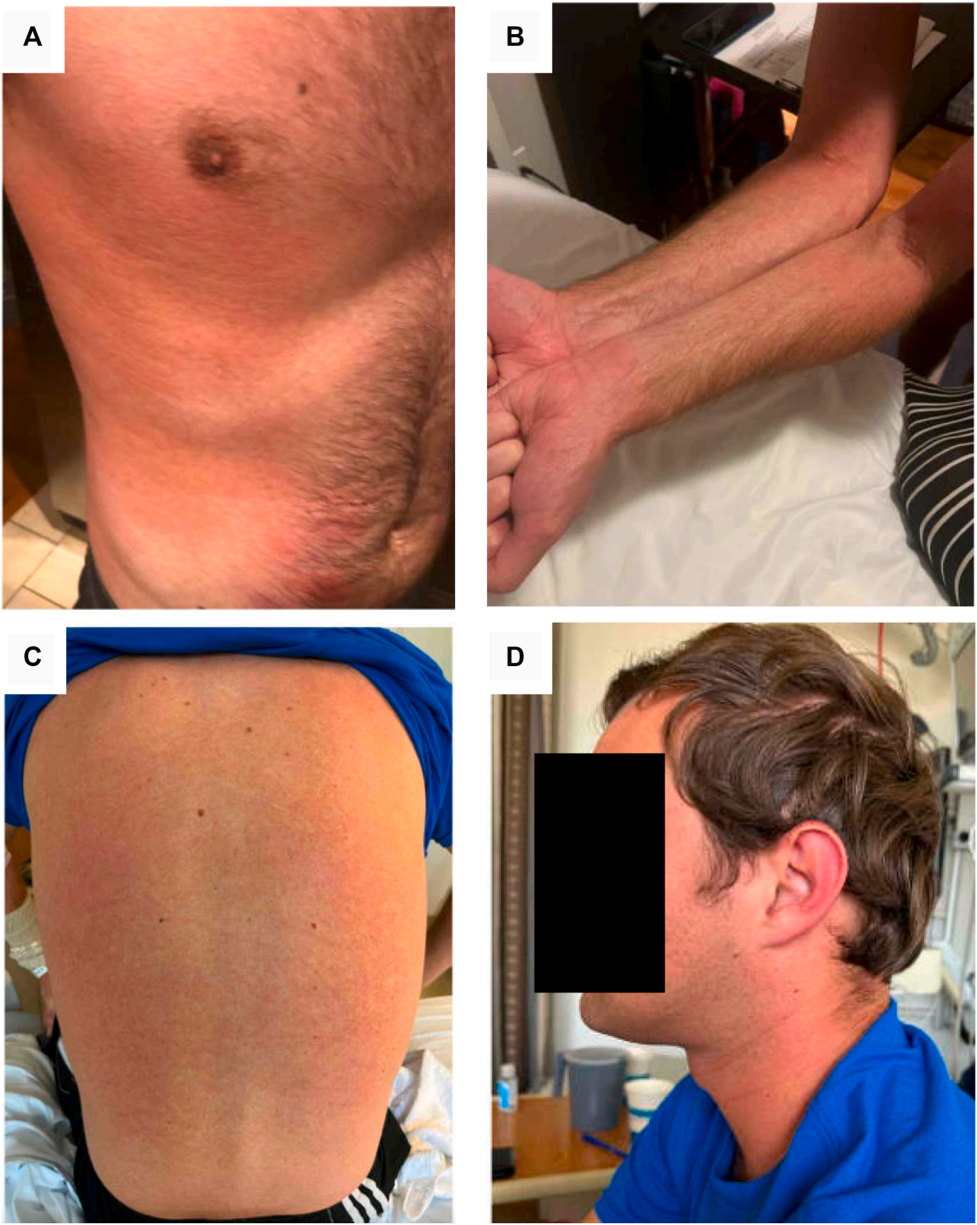
Evolving morbilliform eruption involving the trunk and extremities **(A** and **B)** with progression to confluent erythema with facial involvement **(C** and **D)** despite escalating immunosuppression including anakinra. Biopsy was obtained from the erythematous patch—thin plaque of left flank in panel **(C)**.

A 30-year-old otherwise healthy male without preceding prescription medication exposures presented to the emergency department with daily fevers lasting over a week, sore throat, and asymptomatic, well-defined erythematous urticarial papules and plaques on the extremities (Fig 1, *A* and *B*). Leukocytosis (>27 K/μL, >90% neutrophils) and liver test abnormalities (ALT and AST >120 U/L) were appreciated, though infectious workup was negative. After empiric antibiotics and hospital admission, rheumatology diagnosed the patient with AOSD by Yamaguchi’s criteria^1^ and initiated prednisone 60 mg daily, leading to rash clearance. The following month, the patient was readmitted for fevers to 104 °F, arthralgias, and laboratory derangements (notably ferritin >74,000 ug/L) concerning for AOSD flare with progression to macrophage activation syndrome (MAS). His urticarial eruption recurred and further involved his trunk (Fig 1, *C* and *D*). Following pulses of intravenous methylprednisolone, his home immunosuppression regimen was escalated to oral methylprednisolone 48 mg daily, anakinra 100 mg subcutaneously twice daily (BID), and cyclosporine 100 mg BID. The patient continued to fever and a pruritic, coalescing skin eruption emerged (Fig 2, *A* and *B*), prompting admission to a tertiary referral hospital.

**Fig 3:**
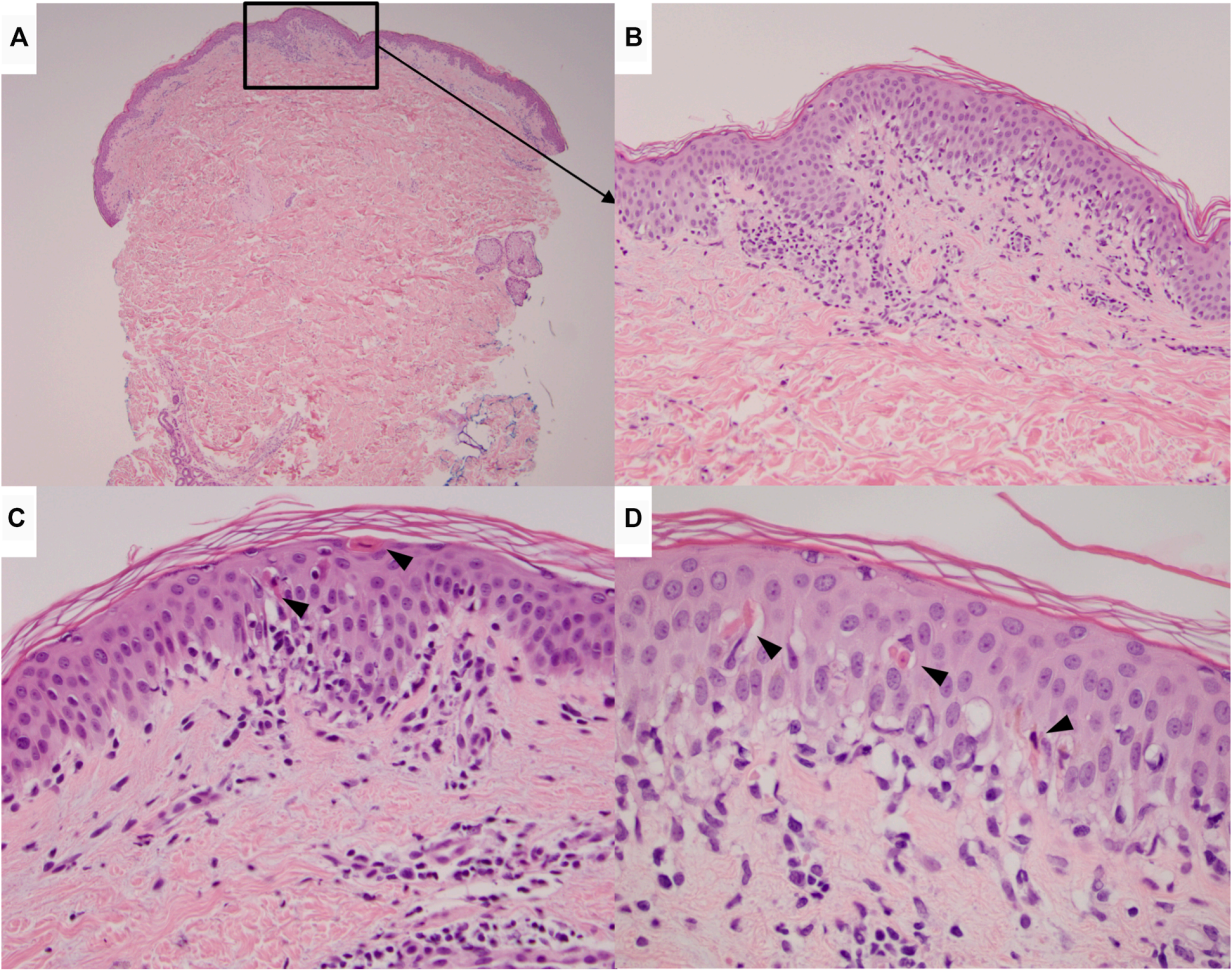
Histopathologic features favoring a drug hypersensitivity reaction. Scanning view of a representative section from punch biopsy of the left flank reveals foci of interface dermatitis **(A,** H&E, ×400**)**, with basal vacuolar changes secondary to a lymphocytic inflammatory infiltrate on higher power view of most prominent focus **(B**, H&E, ×200**)**, additional sections show dyskeratotic keratinocytes (*arrowheads*) spanning all levels of the epidermis **(C** and **D,** H&E, ×400**)**. Rare eosinophils were noted in the dermis (not shown). Concurrent medications at time of biopsy were anakinra, methylprednisolone, pantoprazole, and ruxolitinib.

**Fig 4:**
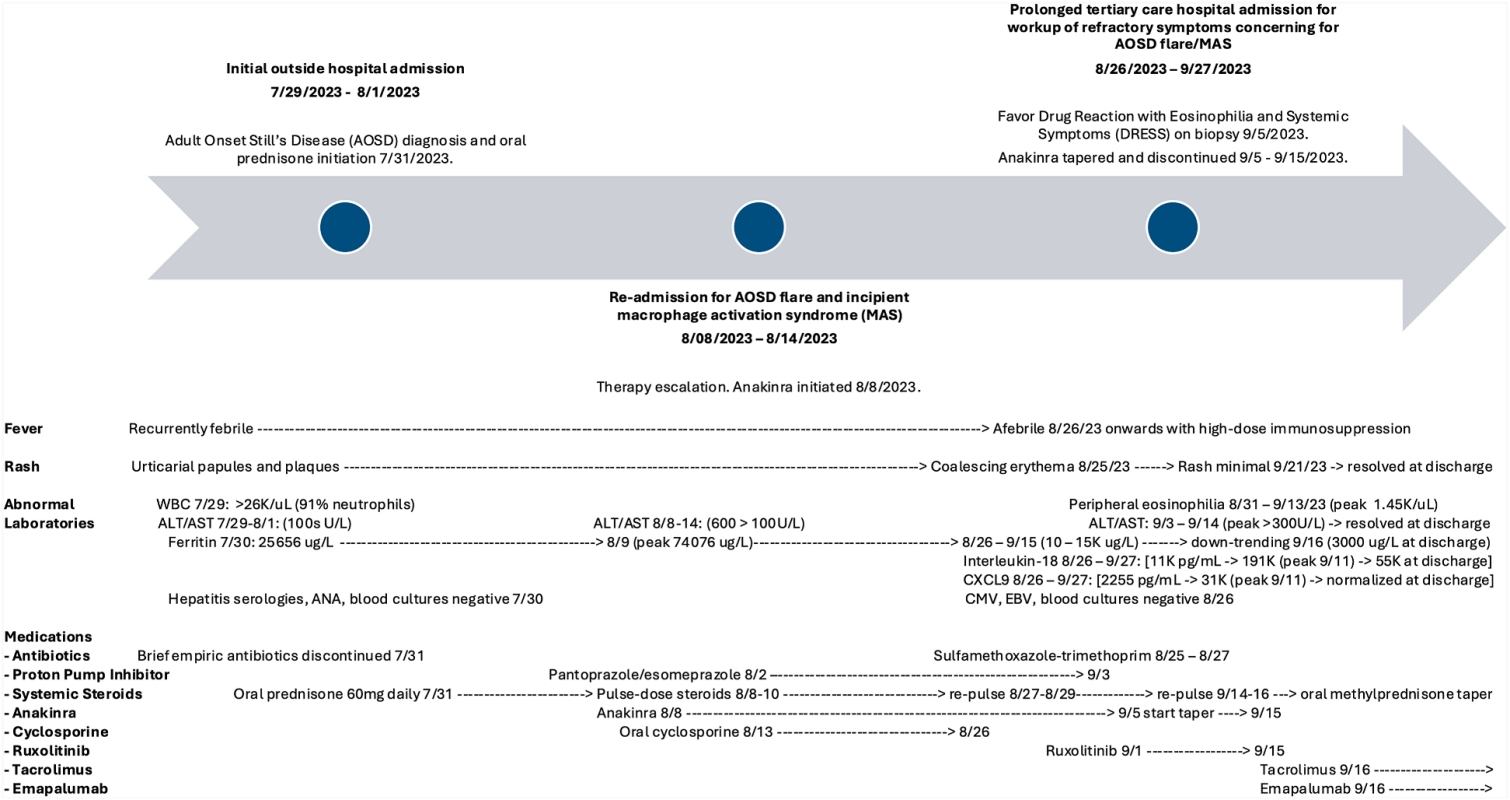
Case timeline highlighting relevant clinical features, laboratory values, and medication exposures.

Further pulse-dosed corticosteroids and a trial of intravenous anakinra (100 mg 4 times daily) did not yield improvement. The patient’s laboratories identified a new, progressive peripheral eosinophilia, rising transaminitis, and elevated IL-18 and CXCL9 (markers for monitoring AOSD/MAS activity, also increased in other inflammatory states). Dermatology was consulted to evaluate for alternative etiologies. A fixed, confluent morbilliform eruption was observed on the trunk and face (Fig 2, *D*). This morphology and associated laboratories raised suspicion for concomitant drug reaction with eosinophilia and systemic symptoms (DRESS). A skin punch biopsy of the left flank (Fig 2, *C*) revealed acute lymphocyte-mediated interface dermatitis with scattered dyskeratosis throughout the epidermis and rare eosinophils in the superficial epidermis (Fig 3). These findings favored a drug hypersensitivity reaction over a primary manifestation of AOSD, and increased the RegiSCAR score from ≥3 (possible) to ≥4 (probable) DRESS. Anakinra was identified as the most likely culprit based on rash onset and duration relative to medication exposures (Fig 4), so formal drug causality testing was not pursued. The patient continued methylprednisolone and transitioned from anakinra to emapalumab, a monoclonal antibody that neutralizes interferon gamma, for maintenance AOSD treatment. Anakinra withdrawal led to resolution of the rash and DRESS laboratory derangements (Fig 4).^3^

## Discussion

The breadth of cutaneous manifestations in AOSD requires clinicians engage in careful observation and a comprehensive diagnostic approach. The classic AOSD rash is an asymptomatic, evanescent salmon-pink maculopapular eruption sparing the face.^1^ However, this narrow concept risks missed diagnoses in skin of color and in cases of atypical eruptions. These atypical AOSD eruptions include urticarial-lichenoid persistent plaques, with a prevalence as high as 65% of AOSD cases, and rarer instances of angioedema, erythroderma, and acneiform lesions.^1^^,^^4^ Distinguishing whether an atypical AOSD eruption is secondary to AOSD or another etiology (eg hypersensitivity or other drug reaction) is critical for appropriate management.

Though the classic AOSD rash can support a diagnosis of AOSD alongside additional clinical criteria,^2^ histopathology can play a pivotal role in the evaluation of atypical rashes with AOSD on the differential. Recent literature highlights dyskeratosis limited to the upper epidermis, often associated with parakeratosis and relatively uninvolved dermal-epidermal junction, as histopathologic features specific to the persistent, localized papules and plaques in AOSD.^4^, ^5^, ^6^, ^7^ Identification of this stratified dyskeratosis could prevent diagnostic delays in patients with this atypical AOSD morphology. Histopathology can also discern main differentials invoked in potential AOSD cases, including connective tissue disease and systemic drug hypersensitivity such as DRESS. DRESS can display distinct histologic findings from classic or atypical AOSD such as eosinophils, generalized interface dermatitis, and multi-level keratinocyte necrosis.^8^

Our case illustrates the potential for DRESS to mimic an AOSD flare; it is a reminder to consider DRESS when faced with therapeutic non-response or atypical clinical findings, and leverage dermatopathology to support this diagnosis and motivate de-escalation of AOSD treatment. Expanding use of targeted therapies in AOSD^2^ has yielded mounting reports of DRESS secondary to IL-1 (anakinra, canakinumab) and IL-6 (tocilizumab) blockade in patients with AOSD – especially those with HLA-DRB1∗15 alleles—as characterized by peripheral eosinophilia, transaminitis, and a fixed morbilliform rash that resolves upon discontinuation of the culprit inhibitor.^3^^,^^9^ Without drug discontinuation, Still’s-DRESS is associated with increased mortality and morbidity, including higher rates of MAS and protracted systemic steroid courses. In our patient, transition from anakinra to emapalumab resulted in full resolution of inflammatory signs and symptoms. Emapalumab, via neutralization of IFN-γ, may be particularly well suited as a bridge therapy for AOSD in Still’s-DRESS patients with concern for MAS.^10^ Of note, while the Still-DRESS population is enriched for HLA-DRB1∗15 alleles, 20% of cases do not have this genetic risk factor, including our patient.^3^ As such, identifying features of Still-DRESS via histology can reassure clinicians in pursuing alternate—and optimal—treatments.

Overall, atypical cutaneous manifestations in AOSD, whether at AOSD onset or further into disease course, warrant skin biopsy for clinicopathologic correlation. Clinicians must remain vigilant for DRESS and other systemic drug hypersensitivities in AOSD patients with generalized, fixed rashes refractory to IL-1 or IL-6 blockade. Additional studies are needed to delineate the scope and clarify the pathogenesis of atypical rashes in AOSD.

## Conflicts of interest

Dr Kumar reports receiving personal fees (advisory boards) from Boehringer Ingelheim, MJH Life Sciences, and SUN Pharma for work unrelated to the current submission. The other authors have no disclosures to declare.
